# Identifying molecular subtypes related to clinicopathologic factors in pancreatic cancer

**DOI:** 10.1186/1475-925X-13-S2-S5

**Published:** 2014-12-11

**Authors:** Shinuk Kim, Mee Joo Kang, Seungyeoun Lee, Soohyun Bae, Sangjo Han, Jin-Young Jang, Taesung Park

**Affiliations:** 1 Department of Statistics, Seoul National University, Seoul, Republic of Korea; 2 Department of Surgery, Seoul National University College of Medicine, Seoul, Republic of Korea; 3 Department of Computer software Engineering, Sangmyung University, Cheonan, Republic of Korea; 4 Department of Mathematics and Statistics, Sejong University, Seoul 143-747, Republic of Korea; 5 Bioinformatics Tech. Lab, Healthcare group, Future Technology R&D Division, SK telecom, Seoul, Korea

## Abstract

**Background:**

Pancreatic ductal adenocarcinoma (PDAC) is one of the most lethal tumors and usually presented with locally advanced and distant metastasis disease, which prevent curative resection or treatments. In this regard, we considered identifying molecular subtypes associated with clinicopathological factor as prognosis factors to stratify PDAC for appropriate treatment of patients.

**Results:**

In this study, we identified three molecular subtypes which were significant on survival time and metastasis. We also identified significant genes and enriched pathways represented for each molecular subtype. Considering R0 resection patients included in each subtype, metastasis and survival times are significantly associated with subtype 1 and subtype 2.

**Conclusions:**

We observed three PDAC molecular subtypes and demonstrated that those subtypes were significantly related with metastasis and survival time. The study may have utility in stratifying patients for cancer treatment.

## Background

PDAC has high propensity for local invasion and early development of metastasis, resulting poor long-term survival [1-3]. Moreover, more than 80% of patients are diagnosed at advanced stages and their survival times are extremely shorter than those from other solid tumors [1]. According to recent reports [4,5], PDAC is the 4^th ^most common cancer accompanied by 4^th ^highest mortality rate among gastrointestinal tract cancers in the U.S.A. [4,5]. In contrast with outcome of treatments improving in other solid cancers, prognosis of pancreatic cancer still remains low and unchanged for the past 15 years. At present, overall median survival of PDAC patients is 13 months, and median survival after R0 resection is 23 months [3].

Conventionally known PDAC factors are difficult to suggest prognostic factors of pancreatic cancer, because those factors are in the depth of invasion, lymph node metastasis, and histologic differentiation. Presently, the prognosis and treatment plan for patients are determined according to these prognostic factors and American joint committee on cancer (AJCC) tumor staging [6]. However, patients with the same AJCC stage or other pathologic prognostic factors have various clinical courses and prognosis. In addition, their responses to chemotherapy vary widely; therefore, established treatment plan and prognosis prediction with molecular datasets should extend patients' survival time. In the same context, identification of molecular subtypes would contribute the comprehensive understanding of a genomic transition and cancer development [1]. Unlike other solid tumor studies, identifying the molecular subtypes of PDAC has been frustrating due to lack of tumor specimens for such studies [2]. We attempted to resolve this problem with surgically collected 106 samples from the Seoul National University Hospital. Identification of molecular subtypes provides stratification of patients by their cancer genome context.

## Materials and methods

### Materials

From 2009 to 2011, 106 patients underwent surgery for pancreatic ductal adenocarcinoma at Seoul National University Hospital approved by the Institutional Review Board. Clinicopathologic data were prospectively collected in electronic medical record form. The patients had a postoperative follow-up for at least 1 year. All of the patients had fresh frozen tissue and acceptable quality of DNA extracted from the tissue. After the operation, 5x5 mm sized tumor tissues were immediately collected from surgical specimens and stored in a -70°C liquid nitrogen tank until DNA extraction. Routinely processed 4-μm thick paraffin-embedded sections from the same lesion were stained with hematoxylin and eosin, then submitted for histologic examination. Concentration of the DNA was calculated with spectrophotometer, and the DNA purity and integrity were evaluated by optical density 260/280 ratio for quality control. We selected 96 samples, which passed quality control test.

### Methods

Survival after resection is associated with many clinical factors such as stage, grade (cell differentiation), and metastasis [7,8]. We extracted 20,219 unique genes out of 22,077 genes (from 33,297 of probe ID) for each sample. All microarray gene expression data sets were transformed to log2 scale. To identify PDAC molecular subtypes, we used consensus clustering methods [9] and non-negative matrix factorization methods (NMF) [10]. Here we mainly discussed NMF clustering algorithm; NMF method is using factorizing expression profiles based on positive matrices decomposition. Main concept of NMF is using the mRNA expression matrix  A( n: genes by  m: subjects) following:

A~WH

where  A is  n by  m matrix, the size of matrix  W is  n by  k, and the size of  H is  k by  m.  k is the column length of  W and same as the row length of  H. The number of clusters is  k as well. To converge to the cost function of NMF algorithm, divergence method is used

(1) $$D\left(A||WH\right)=\underset{ij}{\sum }\left(A_{ij}\text{log}\frac{A_{ij}}{{\left(WH\right)}_{ij}}-A_{ij}+{\left(WH\right)}_{ij}\right)$$

The updating rules for matrix  W and  H are followed by,

(2) $$W_{ia}<-W_{ia}\frac{\underset{u}{\sum }H_{au}A_{iu}/{\left(WH\right)}_{iu}}{\underset{v}{\sum }H_{av}}$$

(3) $$H_{au}<-H_{au}\frac{\underset{i}{\sum }W_{ia}A_{iu}/{\left(WH\right)}_{iu}}{\underset{k}{\sum }W_{ka}}$$

The components of matrix  W and  H are called metagenes, which contain sample and gene information of  A, since those components are related with all of the gene expression levels of samples. Moreover, components of  H contain all gene expression information as well as samples' clustering patterns. More details are explained in [10]. To perform NMF algorithm, we first downloaded Multi experimental view (MeV) [11] from http://www.tm4.org/ Then we used the following parameters and options for MeV's setting: divergence is used for cost function (eq 1) with update rules (eq 2, 3), exponential scale is used for adjusting given data, and maximum iteration is at 1000. Cophenetic correlation coefficient [12] c provides a scalar value measuring robustness across the consensus matrix, by using microarray expression levels for each cluster. The cophenetic correlation coefficients are obtained as 0, being poorly-clustered, to 1, being well-clustered, and calculated by following equation,

$$c=\frac{\underset{i<j}{\sum }\left(Y_{ij}-y\right)\left(Z_{ij}-z\right)}{\sqrt{\underset{i<j}{\sum }{\left(Y_{ij}-y\right)}^{2}\underset{i<j}{\sum }{\left(Z_{ij}-z\right)}^{2}}}$$

Where *Y_ij _*= |*Y_i _*− *Y_j _*| is a distance between two observations;  i,  j, and $Z_{ij}$ is the dendrogrammatic distance of subtype distance between model $Z_{i}$ and $Z_{j}$. The highest value of the cophenetic correlation coefficient determines the optimal number of clusters.

## Results

Demographics and pathologic characteristics of the ninety six pancreatic cancer patients are summarized in Table 1. The mean age of resection for the patients was 65.2 years and the ratio of male to female was 1:1.04. The median for follow up after resection was 14.3 months, and fifty eight patients had recurrence at the end of their follow-ups. R0 resection rate of the patients was 79.2%. Most patients, 94.8%, were diagnosed at Stage II.

**Table 1:** Demographics and pathologic characteristics of the study subjects.

	N = 96
Age (mean ± SD)	65.2 ± 9.1
Sex (M:F)	1:1.04
Operation	
Whipple's operation	20 (20.8%)
PPPD	39 (40.6%)
Distal	30 (31.3%)
Total	6 (6.3%)
Adjuvant chemotherapy	87 (90.6%)
Gemcitabine	56 (58.3%)
5-FU	21 (21.9%)
Unknown	10 (10.4%)
Recurrence	58 (60.4%)
Local	23 (24.0%)
Distant	50 (52.1%)
Follow up (median, months)	14.3 (range, 2.9-45.9)
R status	
R0	76 (79.2%)
R1	14 (14.6%)
R2	6 (6.3%)
AJCC 7^th ^staging	
Stage IA	3 (3.1%)
Stage IB	0
Stage IIA	41 (42.7%)
Stage IIB	50 (52.1%)
Stage III	1 (1.0%)
Stage IV	1 (1.0%)
Histologic differentiation	
Well differentiated	3 (3.1%)
Moderately differentiated	84 (87.5%)
Poorly differentiated	9 (9.4%)
Perineural invasion	83 (86.5%)
Endolymphatic invasion	39 (40.6%)
Endovenous invasion	25 (26.0%)

### Identifying 3 molecular subtypes of PDAC

**Figure 1:**
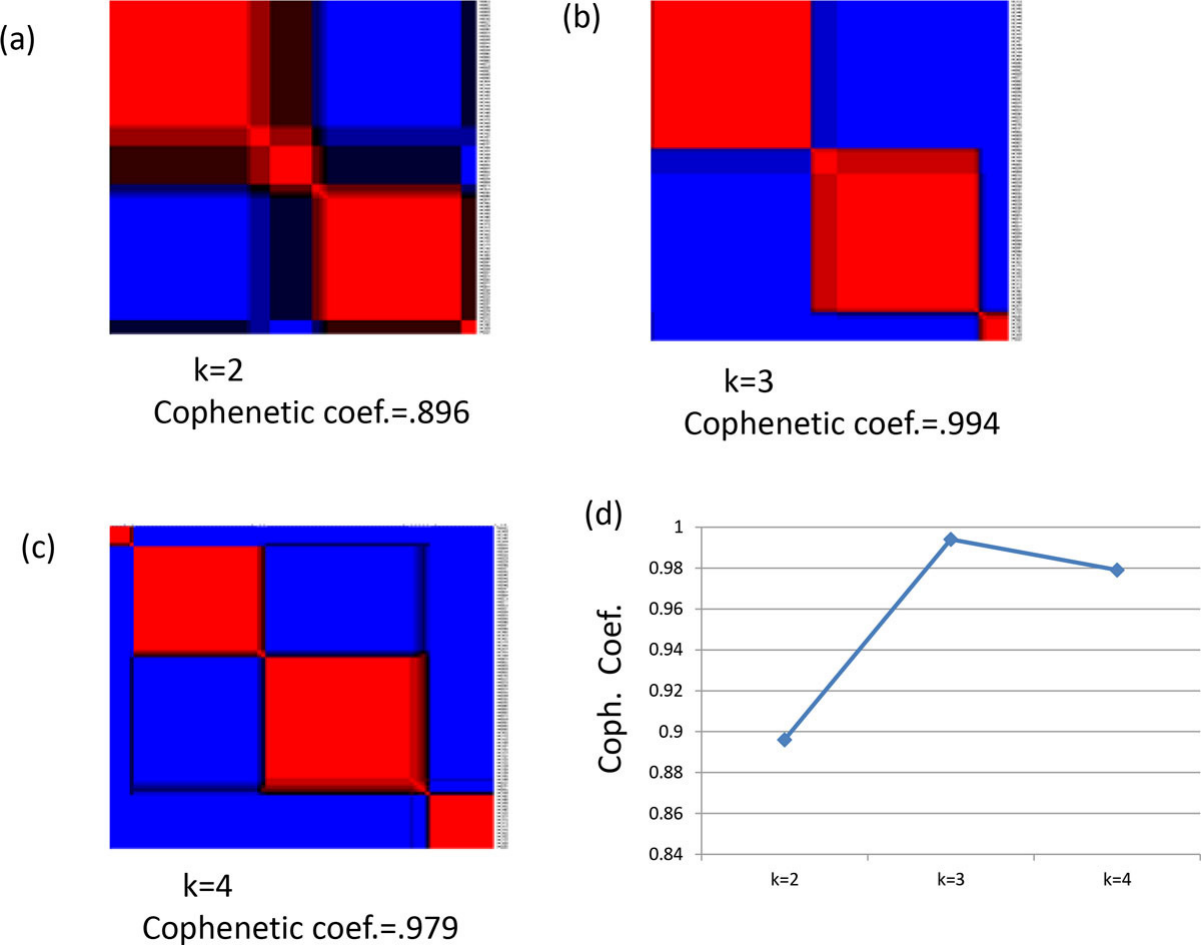
**Plot of NMF performances and Cophenetic coefficients correlation**. (a) k=2 (b) k=3 and (c) k=4 where  k is number of clusters. (d) Illustration of Cophenetic coefficients for number of clusters.

We performed NMF with cophenetic coefficients testing size of cluster from 2 to 4. The resulting cophenetic correlation coefficient for clusters 2, 3, and 4 were 0.896, 0.994, and 0.979, respectively. Figure 1 shows the results of NMF and cophenetic correlation coefficients. Since the maximum peak of the cophenetic correlation coefficients' plot determines the optimal number of subtypes, selecting 3 cluster provides the best separations compared to the rest. Therefore, we further analyzed these 3 groups. In the case of three subtypes, the number of samples for each cluster is 43, 45, and 8 with 27, 22, and 4 censored samples, successively.

### Analysis and comparison between determined subtypes

**Figure 2:**
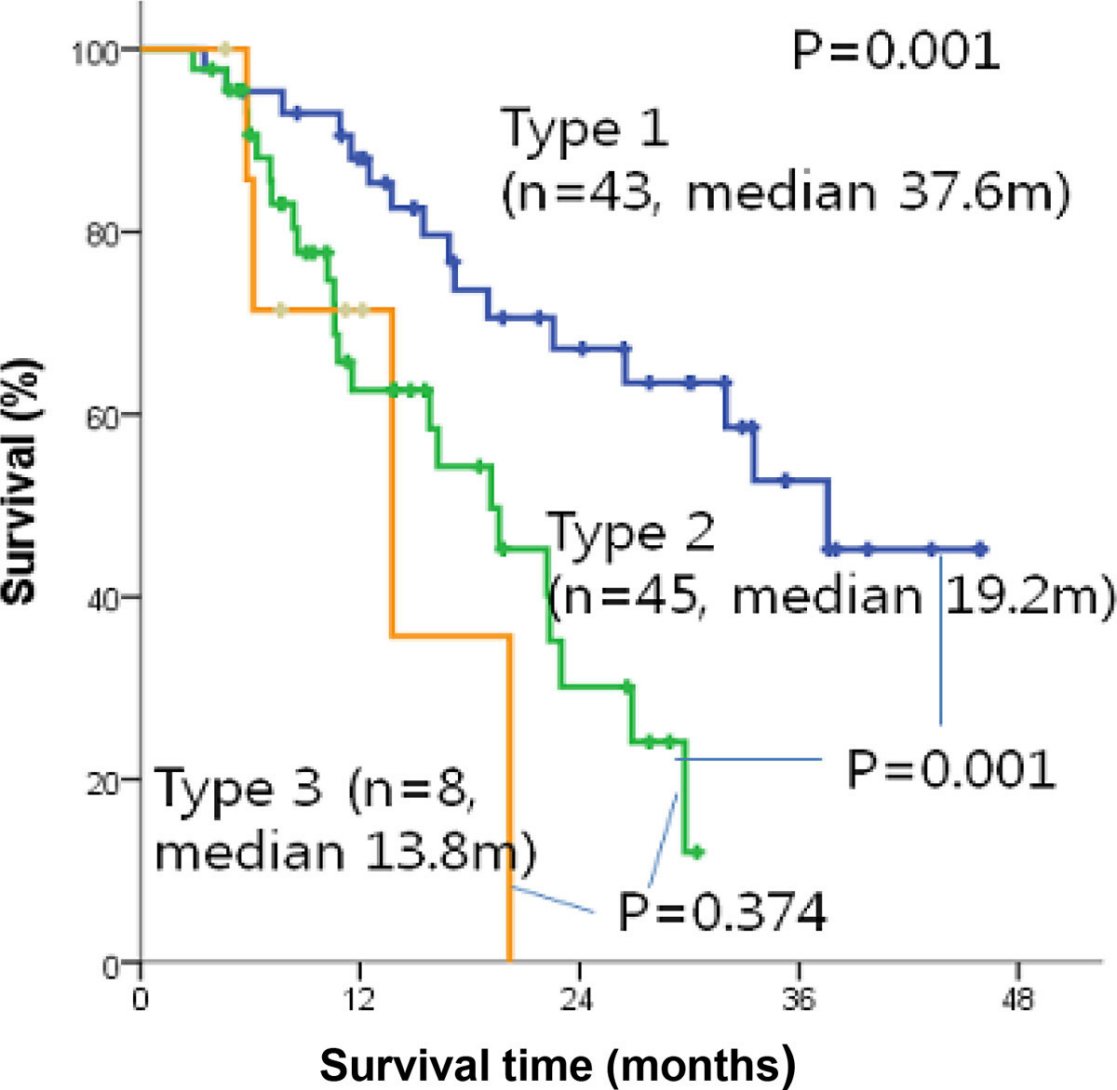
**Kaplan-Meier survival curve**. Kaplan-Meier survival curve comparing survival of individuals with subtype 1 (blue), subtype 2 (green), and subtype 3 (orange) with 0.001 p-value by log-rank statistics test.

We plotted the Kaplan-Meier survival curve using IBM SPSS statistic 20 in Figure 2. The median of overall survival was 23 months, while the median survival times are 37.6, 19.2, and 13.8 months for each subtypes 1, 2, and 3, respectively. The p-value from the log-rank test comparing subtypes 1 and 2 is 0.001, comparing subtypes 1 and subtype 3 is 0.008, while the p-value between subtypes 2 and 3 is 0.374. Consistently, longer surviving patients have much less metastasis disease according to Table 2. Although subtypes 2 and 3 are clearly separated in NMF, with cophenetic correlation coefficients 0.97, Kaplan-Meier curve is not statistically significant; this might be due to the small sample size of subtype 3. Comparison results of clinicopathologic characteristics according to 3 molecular subtypes are summarized in Table 2.

**Table 2:** Clinicopathologic characteristics according to 3 molecular subtypes.

	Subtype 1 (n = 43)	Subtype 2 (n = 45)	Subtype 3 (n = 8)	P-value
Age (mean ± SD)	66.3 ± 8.1	64.2 ± 10.3	64.8 ± 7.4	0.570
Male gender	22 (51.2%)	20 (44.4%)	5 (62.5%)	0.585
Tumor size (cm)	3.0 ± 1.0	3.4 ± 1.1	3.6 ± 0.8	0.073
R status				0.029
R0	39 (90.7%)	31 (68.9%)	6 (75.0%)	
R1, R2	4 (9.3%)	14 (31.1%)	2 (25.0%)	
AJCC Stage				0.304
Stage IIA	19 (44.2%)	21 (46.7%)	1 (12.5%)	
Stage IIB	21 (48.8%)	22 (48.9%)	7 (87.5%)	
Histologic differentiation				0.417
Well differentiated	1 (2.3%)	1 (2.2%)	1 (12.5%)	
Moderately differentiated	39 (90.7%)	38 (84.4%)	7 (87.5%)	
Poorly differentiated	3 (7.0%)	6 (13.3%)	0	
Perineural invasion	35 (81.4%)	40 (88.9%)	8 (100%)	0.298
Endolymphatic invasion	17 (39.5%)	18 (40.0%)	4 (50.0%)	0.690
Endovenous invasion	9 (20.9%)	16 (35.6%)	0	0.070
Adjuvant chemotherapy	37 (86.0%)	43 (95.6%)	7 (87.5%)	0.215
Gemcitabine	21 (48.8%)	30 (66.7%)	5 (62.5%)	0.267
5-FU	10 (23.3%)	10 (22.2%)	1 (12.5%)	0.936
Unknown	6 (14.0%)	3 (6.7%)	1 (12.5%)	
Recurrence	21 (48.8%)	32 (71.1%)	5 (62.5%)	0.091
Local	10 (23.3%)	11 (24.4%)	2 (25.0%)	1.0
Distant	17 (39.5%)	30 (66.7%)	3 (37.5%)	0.022

The mean age of each cluster is 66.3 (± 8.1), 64.2 (± 10.3), and 64.8 (± 7.4) for subtype 1, subtype 2, and subtype 3, respectively. R0 resection rate was significantly higher in subtype 1 (p = 0.029) than in subtypes 2 and 3.

Tumor size tended to be larger (p = 0.073) and endovenous invasion rate lower (p = 0.070) in subtype 3 than in subtypes 1 and 2. Recurrence rate inclined to be lower in subtype 1 than in subtypes 2 and 3 (p = 0.091) and distant metastasis rate tended to be higher in subtype 2 than in subtypes 1 and 3 (p = 0.022).

Especially, when comparing only subtype 1 with subtype 2, the ratio of R0 was significantly higher in subtype 1 than in subtype 2 (90.7% vs. 68.9%, p = 0.016), and distant metastasis ratio to non-metastasis was significantly higher in subtype 2 than in subtype 1 (66.7% vs. 39.5%, p = 0.018). The prognosis of subtype 2 is significantly worse than subtype 1 (median 19.2 vs. 37.6 months, p = 0.001). However, average age, sex, and local invasion are not significant among subtypes with p >0.5. The result implies that these molecular subtypes are useful for poor-prognosis markers for cancer treatment, by triggering the target genes.

### Analysis and comparison of subtype 1 and subtype 2 restricted to R0 resection patients

**Figure 3:**
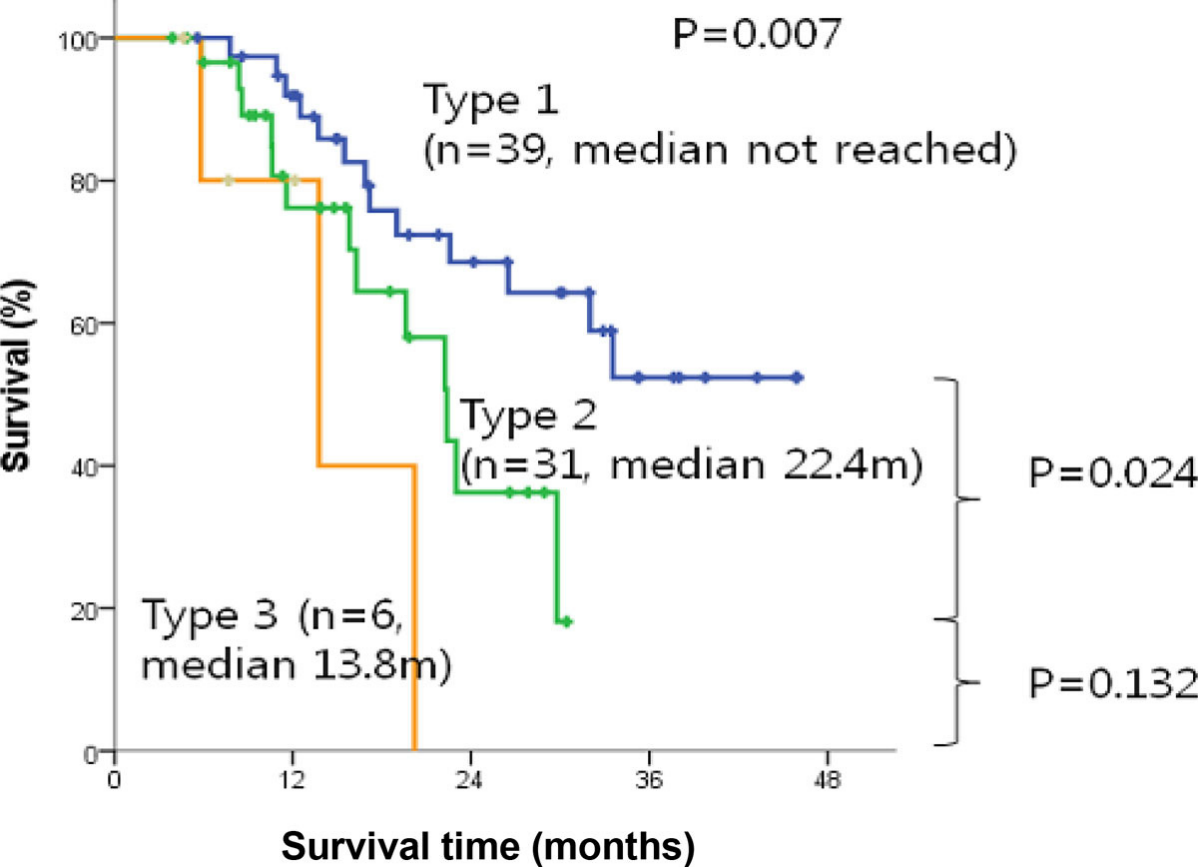
**Kaplan-Meier survival curve for R0 resection with survival**.

**Figure 4:**
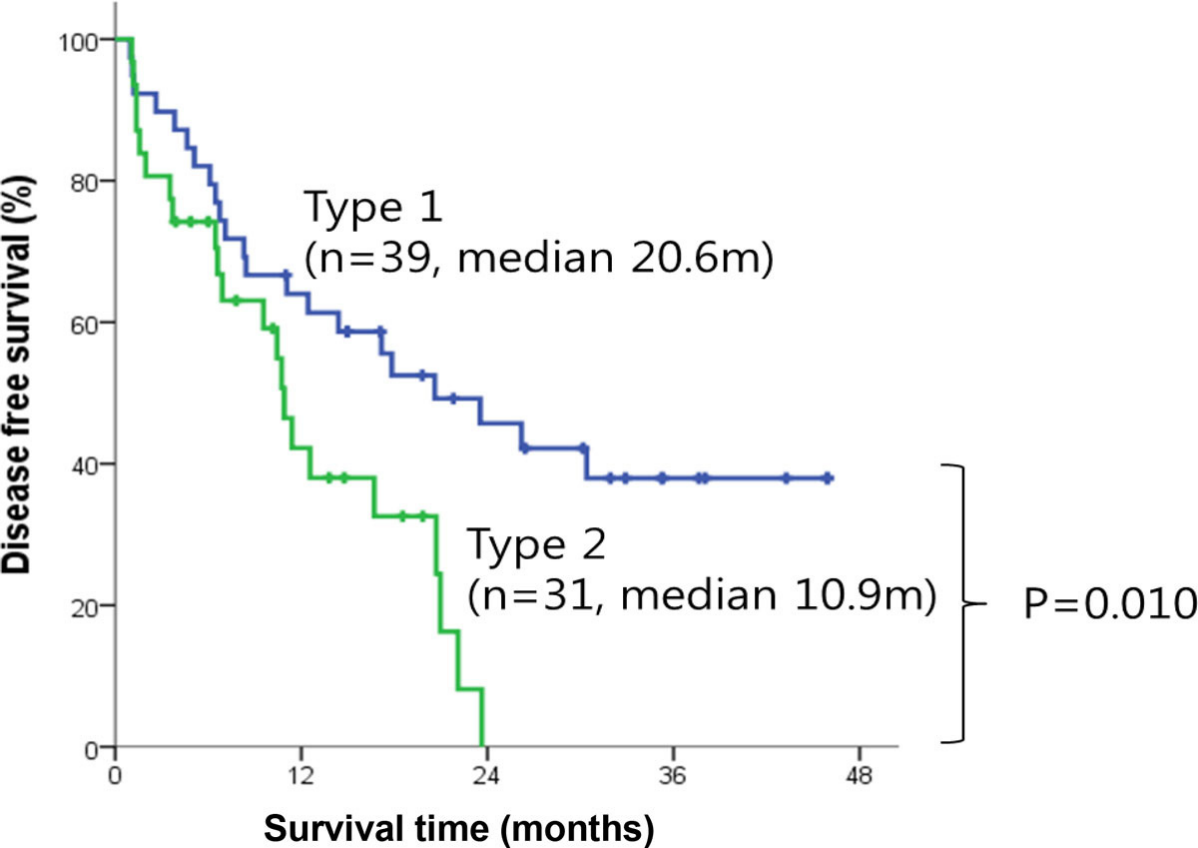
**Kaplan-Meier survival curve for R0 resection with disease free survival**.

We also analyzed sub-clinicopathologic characters restricted to R0 resection between 39 subtype 1 patients and 31 subtype 2 patients. Metastasis is significant between subtype 1 (n = 15, 38.5%) and subtype 2 (n = 21, 67.7%) with p-value = 0.018. We plotted Kaplan-Meier curve with R0 resection survival time demonstrating that prognosis is significantly poor in subtype 2 (median 22.4 mo) compared to subtype 1 (median not reached) with p = 0.024 in Figure 3. Disease free survival was significantly lower in subtype 2 than that of subtype 1 (median 10.9 vs. 20.6 months, p = 0.010) in Figure 4.

### Identifying enriched pathway between subtype 1 and subtype 2

For functional assessment of our subtype identification, we performed gene set enrichment analysis [13] to get enriched pathway information for subtype 1 and subtype 2. Since sample size of subtype 3 is much smaller than that of the other two, we excluded subtype 3 in this analysis. In this step of the analysis, we used KEGG pathway with 200 individuals downloaded from the molecular signatures data base (MSigDB) [13] with 1,000 permutations. The results of top nine pathways ordered by their absolute normalized enriched score in each subtype, are shown in Table 3. FDR q-value of all enriched pathways is less than 0.25.

**Table 3:** Enriched pathways of subtype 1 and subtype 2.

Enriched pathway in Subtype 2 (poor-prognosis)	Enriched pathway in Subtype 1 (good-prognosis)
HSA03010_RIBOSOME	HSA04080_NEUROACTIVE_LIGAND_RECEPTOR_INTERACTION
HSA00190_OXIDATIVE_PHOSPHORYLATION	**HSA04060_CYTOKINE_CYTOKINE_RECEPTOR_INTERACTION **
HSA04520_ADHERENS_JUNCTION	HSA04742_TASTE_TRANSDUCTION (Sensory system)
HSA04510_FOCAL_ADHESION	**HSA04020_CALCIUM_SIGNALING_PATHWAY **
HSA03050_PROTEASOME	**HSA04640_HEMATOPOIETIC_CELL_LINEAGE (Immune system) **
HSA05212_PANCREATIC_CANCER	HSA00140_C21_STEROID_HORMONE_METABOLISM
HSA04120_UBIQUITIN_MEDIATED_PROTEOLYSIS	HSA00940_PHENYLPROPANOID_BIOSYNTHESIS
HSA05211_RENAL_CELL_CARCINOMA	HSA01430_CELL_COMMUNICATION
HSA05220_CHRONIC_MYELOID_LEUKEMIA	HSA00430_TAURINE_AND_HYPOTAURINE_METABOLISM

The enriched pathways of subtype 2 were related with fatal disease pathways including pancreatic cancer, renal cell carcinoma, and chronic myeloid leukemia. On the other hand, the enriched pathways of subtype 1 were related to immune system, such as hematopoietic cell lineage, cytokine-cytokine receptor interaction, and calcium signaling pathway. The findings of enriched pathways in Table 3 are consistent with survival analysis in Figure 1.

### Identifying significant biomarkers for each subtype

**Figure 5:**
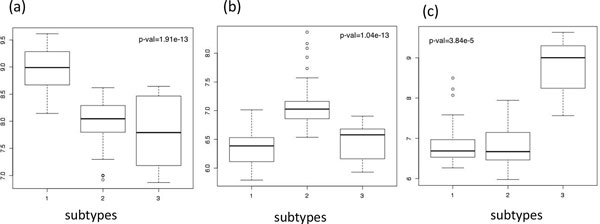
**Boxplots of Kruskal-Wallis test using overexpressed genes in each subtype**. Boxplots of Kruskal-Wallis test for comparing 3 subtypes (a) using overexpressed genes of subtype 1, (b) using overexpressed genes of subtype 2, and (c) using overexpressed genes of subtype 3.

More importantly, we identified differentially expressed genes between subtypes using significant analysis of microarray (SAM) [14]. Significant genes specific to each group were chosen one versus the rest, which implies one group (subtype 1) is compared to the other two groups (subtypes 2 and 3). Boxplot with Kruskal-Wallis test supported our clustering in Figure 5. We selected top 20 genes with 0 q-value ordered in fold-change for up-regulated genes in case subtypes, in Table 4. 10 bold genes were also found by Collisson [2] as PDAC assigner genes among 62. Interestingly, the 9 highlighted genes in subtype 3, on Korean pancreatic subtypes, are found at exocrine-like subtype assigner genes in three identified subtypes in Figure 1 (a) of previous study [2]. However, the proportion of sample size of subtype 3 from total Korean PDAC patients are much smaller than that of Exocrine-like subtype from GSE15471 data sets nm2344-S2 in [2], which are 8% and 36%, respectively. This excessive difference, between the two datasets of equal subtypes, require an extended study in the future.

**Table 4:** Significant genes for each subtype, 10 bold genes were also identified by Collisson [2].

Subtype1	Subtype2	Subtype3
LOC100132217	SLC6A14	**CTRB2**
REXO1L2P	**CKS2**	**PLA2G1B**
REXO1L1	DSG2	**GP2**
LOC349196	KLK6	ALDOB
USP17L6P	ERO1L	CTRB1
KRTAP10-4	ANXA1	**CELA3B**
FAM90A18	SC4MOL	**CLPS**
KRTAP4-9	SLCO1B3	CTRC
NCRNA00268	TMPRSS4	**PRSS2**
FAM90A10	MET	**REG3A**
FAM90A20	PTPN12	ANPEP
KRTAP4-7	CDK6	TRY6
LOC440570	CSE1L	**REG1A**
USP17		**PNLIPRP2**
GAGE12J		SYCN
FAM90A13		ERP27
C5orf60		CTRL
KRTAP5-7		PNLIPRP1
OR7E125P		GATM
KRTAP4-4		REG3G

### Validation of the results

**Figure 6:**
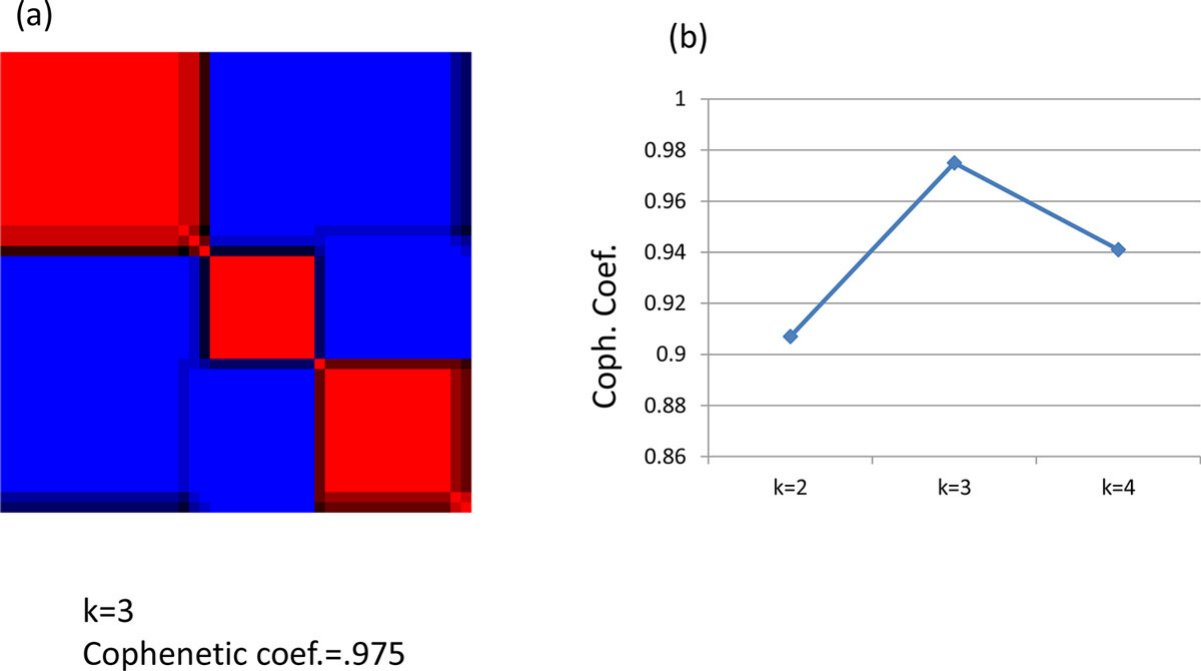
**Plot of NMF performance k = 3 (a), and cophenetic coefficients of validation data sets (b)**.

For the validation study of our findings, we used an independent dataset GSE28735 [15] downloaded from Gene expression omnibus. GSE28735 consists of 45 PDAC samples. We extracted all biomarkers in Table 4 from each validation sample and implemented NMF from rank 2 to rank 4. The highest cophenetic coefficient is 0.975 when rank is 3 in Figure 6. The best result of implementing Kaplan-Meier survival analysis was to compare subtype 3 versus rest, which yielded p-value 0.198. The p-values are 0.384 and 0.522 for the tests comparing subtype 1 versus rest and subtype 2 versus rest, respectively. The significant biomarkers matched 9 out of 13 for subtype 2 and 13 out of 20 for subtype 3 in Table 4.

## Conclusions

It is an important issue to identify molecular subtypes for stratifying PDAC patients depending on clinicopathologic factors and molecular gene expression. These identified molecular subtypes can be utilized for stratifying patients into their appropriate treatment groups. In this regard, we used total of 96 PDAC samples, and identified 3 molecular subtypes which were significantly related to clinicopathologic factors such as metastasis, tumor size, residual, and survival time. The results consistently demonstrate that poor prognosis is significantly related to metastasis. We also identified enriched pathways for poor-prognosis and good-prognosis related to fatal diseases and immune system, respectively, in Table 3. Moreover, we suggested gene markers represented for each subtype to use in PDAC stratification. We also considered the restricted to R0 resection samples in each subtype. Prognosis was significantly worse in subtype 2 than in subtype 1. Disease free survival rate was significantly lower in subtype 2 compared to subtype 1. In addition, 13 out of 22 over-expressed genes of subtype 3 in our findings are also found in exocrine-like subtypes in previous study [2], but further study is required on the radically short survival time for Korean specific biomarker for PDAC subtypes using larger sample size.

Nevertheless, our findings have some limitation for being applied to the patients directly. Even though we selected the significant gene sets using strong machine learning tools, and successfully clustered 3 classes in validation data sets, we still need a further investigation for validation of following up patients and/or using new data sets. At this moment, such high quality gene expression data sets including R0 resection, metastasis and survival time information are not available in public.

## List of abbreviations used

PDAC: Pancreatic ductal adenocarcinoma; NMF: Non-negative matrix factorization methods; AJCC: American joint committee on cancer; MeV: Multi experimental view; SAM: Significant analysis of microarray; MSigDB: The molecular signatures data base.

## Competing interests

The authors declare that they have no competing interests.

## Authors' contributions

SK led the analysis of the data and drafted the manuscript. MJ analyzed the data, collected the data and literature searches, JJ collected the data and conceptualized the project. SH designed and performed microarray experiment. TP & SB contributed to conceptualization of the initial project and TP drafted the manuscript. SL advised the analysis. All authors read and approved the final manuscript.

## Declarations

Publication of this article has been funded by Healthcare Group, Future Technology R&D Division, SK Telecom Co., by the National Research Foundation of Korea (NRF) grant funded by the Korea government (MSIP) (No. 2012R1A3A2026438, 2008-0062618), and by a grant from the National R&D Program for Cancer Control (No. 1120310).

This article has been published as part of *BioMedical Engineering OnLine *Volume 13 Supplement 2, 2014: Selected articles from the IEEE International Conference on Bioinformatics and Biomedicine (BIBM 2013): BioMedical Engineering OnLine. The full contents of the supplement are available online at http://www.biomedical-engineering-online.com/supplements/13/S2.
